# A Cake Made with No Animal Origin Ingredients: Physical Properties and Nutritional and Sensory Quality

**DOI:** 10.3390/foods12010054

**Published:** 2022-12-22

**Authors:** Diana Ansorena, Lucía Cartagena, Iciar Astiasaran

**Affiliations:** 1 Center for Nutrition Research, Department of Nutrition, Food Science and Physiology, Faculty of Pharmacy and Nutrition, University of Navarra, 31009 Pamplona, Spain; 2 IDISNA–Instituto de Investigación Sanitaria de Navarra, 31008 Pamplona, Spain

**Keywords:** vegan, sunflower oil, soy protein, gelled emulsion, bakery product

## Abstract

A gelled emulsion ingredient based on high oleic sunflower oil (20%) and an isolated soy protein suspension were used in the elaboration of a cake to avoid the use of ingredients of animal origin. The control product was elaborated with butter and milk. Sugar was used in both types of formulations, but it was partially replaced by maltitol in the reformulated product. Decreases of 25% in energy and 67% in fat supply were achieved, as well as a 36% reduction in the sugar content. The saturated fatty acid amount was 0.57 g/100 g product, in contrast with the 9.45 g/100 g product found in control products. Differences in color were observed both through instrumental and sensory analysis, especially in the crust, with lower values for the Browning index in the reformulated products. The hedonic test, carried out with 44 untrained panelists, showed a good score for general acceptability (6.1 in contrast to 7.2 for control products), and no significant differences from the control were found for flavor.

## 1. Introduction

Health-related aspects and sustainability are among the most relevant food innovation drivers and lie behind many of the currently made reformulation strategies to ultimately decrease the prevalence of diet-related chronic disease and improve public health [1].

Bakery products are very appreciated by consumers because of their appealing sensorial properties. However, nowadays, dietary guidelines recommend the occasional intake of these products because of their usually high amounts of sugar and fat. A thorough review of the factors influencing the sensory perception of reformulated baked confectionary products pointed out that the raw materials have a major impact on flavor perception, and that the modifications of fat and sugar can have a significant impact on sensory quality [2].

The traditional formulations of bakery products include flour (usually wheat flour), sugar, eggs, fat (usually butter), water (and sometimes milk), and a leavening agent. Sugar contributes to volume and viscosity and stabilizes the emulsion and foam system in the cake batter by increasing the interfacial density among the dispersed droplets [3,4]. However, high sugar intake is strongly associated with the high incidence of several non-communicable diseases, such as cardiovascular disease or diabetes [5]. Also, free sugars are the primary dietary factor responsible for caries and are also important to consider for obesity control. In order to decrease the amount of added sugars, substitution with different sweeteners has been tried. In this sense, it has been pointed out that both the sweetener type and sweetener concentration not only affect the taste of the products but also determine the final set structure and technological properties of baked goods by modifying the starch thermal properties (gelatinization, pasting/swelling, retrogradation) [6,7]. Polyols provide good bulk characteristics for application in bakery products but occasionally have a low relative sweetness, which limits their applicability from a sensory point of view [8]. Danish cookies formulated with the partial replacement of sucrose with up to 50% erythritol had sensory and physical quality characteristics comparable to cookies prepared with 100% sucrose [9].

Fat plays an important role both in the technological and sensorial properties of bakery products. This makes it rather difficult to eliminate or even reduce this ingredient without causing a deleterious impact on the quality of the reformulated product. Consequently, most of the reformulation strategies applied so far have been focused on the use of fat replacers (proteins, carbohydrates, fiber) that mimic fat properties and/or on the substitution of animal fat (butter) for pre-emulsified vegetable oils. These strategies contribute to reducing the final amount of saturated fat (SFA), cholesterol, and calories, which are the three factors with negative health connotations. Functional ingredients based on structured oils, such as emulsions or gelled emulsions made with vegetable oils with bioactive compounds, have great potential to be developed and applied in a wide range of bakery products, thus producing nutritionally enhanced foods and technologically and sensory-acceptable products [10]. These types of ingredients are also easy to apply during processing at an industrial level and have no high economical cost.

Eggs constitute an important ingredient in bakery products. They contribute to the stabilization of emulsion, foam ability, and building up of firm gels [11]. They also supply different nutrients (proteins, fat, cholesterol, liposoluble vitamins and minerals) and carotenoids, which could contribute to the final color of the product. However, recent studies have been focused on using egg replacers due to health-related issues (phenylketonuria, allergy, high cholesterol, avian influenza) and also due to dietary preferences, such as vegan–vegetarian habits or religious beliefs [12]. For instance, the successful replacement of eggs was achieved by using Chubak root extract and guar gum in eggless cake formulation [13]. Milk is another animal-origin ingredient that is sometimes used as an egg replacer [14] and also supplies high nutritional value due to its protein, fat, and micronutrient contents.

The EAT–Lancet Commission on healthy diets from sustainable food systems [15] explained that a healthy and sustainable diet has to be based on vegetables, fruits, whole grains, legumes, nuts, and unsaturated oils; includes a low to moderate amount of seafood and poultry; and includes no or a low quantity of red meat, processed meat, added sugar, refined grains, and starchy vegetables.

It is definite that the current trend toward more sustainable production processes and a healthier diet has given rise to a decrease in the use of animal-origin foods. In this context, the aim of this work was to elaborate a cake with only vegetable ingredients, thus replacing butter and decreasing the added sugars. An emulsion gel made with high oleic sunflower oil, soy protein, and maltitol used as alternative ingredients.

## 2. Materials and Methods

### 2.1. Materials

Wheat flour (75% carbohydrates, 4.0% of which were sugars, 10% protein, 2.0% fat, and 0.03% salt; Gallo, El Carpio, Spain), granulated sugar (Azucarera Iberia, Madrid, Spain), high oleic sunflower oil (Urzante, Tudela, Spain), milk (Central Lechera Asturiana, Siero, Spain), butter (President, Laval, France), apple cider vinegar (Dietisa, Sant Cugat del Vallés, Spain), salt, baking powder (Mondelez, Madrid, Spain), and vanilla extract (Vahiné, McComick España S.A., Sabadell, Spain) were purchased in a local market. Maltitol (Maltidex CH 16385) was obtained from Cargill (San Sebastian, Spain). Polysorbate 80 was obtained from Sigma-Aldrich Chemical Co. (Saint Louis, MO, USA). Alginate, as Binder 1.0 (alginate and calcium sulfate), was obtained from BDF Ingredients (Girona, Spain) and soy protein isolate (90% protein) from T’aliment (Salvador de Guardiola, Spain).

### 2.2. Gelled Emulsion Preparation

The gelled emulsion was elaborated with high oleic sunflower oil (20%), alginate (2%), polysorbate 80 (0.12%), and water (77.8%), following the protocol described by Gutierrez-Luna et al. [16]. Firstly, polysorbate and high oleic sunflower oil were mixed under gentle stirring. This oil phase was added to the water and then mixed with an electric mixer at medium speed until the emulsion formed. Finally, alginate was added to stabilize the emulsion and blended until the sample was a homogeneous mixture. Once the gelled emulsion was formed, it was kept under refrigeration (4 °C) overnight until being used.

### 2.3. Soy Protein Suspension Preparation

Soy protein isolate at 90% was used to obtain a water suspension that would supply, in the reformulated cake, the same amount of protein supplied by the whole milk in the control products. A 6 g aliquot of soy protein isolate was mixed with water and homogenized with an electric mixer until obtaining a homogeneous mixture (100 g of suspension).

### 2.4. Cakes Preparation

The formulations used for the cake’s preparation are shown in Table 1. Whole milk and butter were totally replaced by the gelled emulsion and the soy protein suspension. The amounts of these ingredients were calculated to maintain a similar level of moisture and proteins in the dough between the control and the reformulated product. Sugar was partially substituted by maltitol.

Milk (control) or soy protein suspension (reformulated cake) and apple cider vinegar were mixed and allowed to stand for a few minutes. The dry ingredients, wheat flour, baking powder, maltitol (reformulated cake), and salt were mixed in a large bowl. Butter (control cake) or gel (reformulated cake) was firstly beaten until creamy with an electric hand mixer (ErgoMixx Style MS64M6170, Robert Bosch Hausgeräte GmbH, München, Germany) at medium-high speed for 3 min. Then, the sugar and the vanilla extract were added to the butter or gel, and they were beaten at high speed for 4 min. The dry ingredients (wheat flour, baking powder, maltitol, and salt) and the milk/soy protein mixture were alternately incorporated into the mix (butter/gel + sugar + vanilla) and mixed until homogenization. The control and reformulated batters were placed in silicon pans (20 × 5 cm) and baked in a convection oven (Mod 505, Balay, Zaragoza, Spain) at 180 °C for 55 and 65 min, respectively. The oven was preheated at 180 °C for 20 min. The cakes were kept at room temperature overnight before analysis.

Three different batches of cakes were prepared for each type of product.

### 2.5. Proximate Composition Analysis

Moisture, ash, and protein content were determined according to the official methods 950.46, 920.153, and 928.08, respectively [17,18,19]. Carbohydrates were calculated by difference. Fat was determined using a Soxhlet extractor B-811 Büchi extraction system [20], prior to acid hydrolysis [21].

Every parameter was measured for quadruplicate in each batch.

### 2.6. Fatty Acids Profile

Fatty acids methyl esters (FAME) were prepared using boron trifluoride/methanol [22], and lipid profiles were determined through gas chromatography using a Perkin Elmer Clarus 500 with a flame ionization detector (FID) and a capillary column SP-2560 (100 m × 0.25 mm × 0.2 µm). The injector and detector temperatures were set at 250 and 260 °C, respectively. The oven temperature was set at 175 °C for 10 min, increased to 200 °C at a rate of 10 °C/min, and then increased to 220 °C at a rate of 4 °C/min and held for 15 min. Hydrogen was used as the carrier gas, with a pressure of 30 psi and a split ratio of 120 mL/min. The identification and quantification of the fatty methyl esters were carried out using heptadecanoic acid methyl ester as an internal standard [23].

### 2.7. Color

The color was determined both in the crust and in the crumb using a Chromameter-2-CR-200, Minolta, Osaka, Japan) colorimeter. For crust determinations, the colorimeter was placed on four different locations of the surface per cake. For crumb determinations, the cakes were cut into quarters and the colorimeter was placed at four different locations per cake. L* (brightness), a* (redness), and b* (yellowness) were determined. The hue angle (h) and chrome (C*) were calculated using Equations (1) and (2), respectively.

h (hue angle) = arctan (b*/a*)(1)

C* (chroma) = (a*^2^ + b*^2^) ^(1/2)^ (2)

The Browning Index (BI) was calculated according to Ureta et al. [24] using Equation (3) below:

BI = (100(X − 0.31))/0.172 where X = (a* + 1.75L*)/(5.645L* + a* − 3.012b*)(3)

### 2.8. Texture (Hardness)

Hardness was measured at room temperature using a TA-TXT2 texturometer (Stable Micro Systems, Texture Technologies Corporation, Scarsdale, NY, USA) equipped with a 25 kg load cell capacity and a 75 mm diameter stainless steel flat probe (P/75). Cubes of 2.0 × 2.0 × 2.0 cm, taken from the center of the crumb, were used for texture measurements. A total of 12 cubes were analyzed for each formulation. The samples were compressed to 50% of their original height at a speed of 1.0 mm/s.

### 2.9. Sensory Analysis

A hedonic sensory analysis was carried out with 44 untrained panelists (non-smokers, between 20 and 25 years of age), who were all familiar with the products being tested. Participants were informed of the allergens (Regulation 1169/2011) present in the samples (milk, soy, and gluten) before the analysis. The panelists were divided into two groups, and the two cakes were presented in a different order for each group. A 9-point hedonic scale was used, from 1 (extremely dislike) to 9 (extremely like). The color, flavor, taste, juiciness, and general acceptability were evaluated for both the control and reformulated cakes [25]. Sample portions of 2 × 2 cm were presented on white dishes. Cakes were baked 24 h before the sensory analysis.

### 2.10. Statistical Analysis

Independent samples t-tests were used to determine statistical differences between the control and reformulated samples using SPSS 22 software (IBM SPSS Statistics for Windows, Version 22.0. Armonk, NY, USA: IBM Corp.). Statistical differences were determined at a 95% confidence level.

## 3. Results and Discussion

### 3.1. General Composition and Fatty Acid Profile

A cake made with wheat flour, whole milk, sugar, and butter as the main ingredients was chosen as a control for this work. This product showed a standard nutritional profile for this type of product, with carbohydrates being the main compounds, followed by lipids and proteins (Table 2). The reformulated product showed a lower energy value, reaching a decrease of 24% compared to the control. Although it was a quantitatively high reduction, it was not enough to allow the product to bear the “energy reduced” claim according to UE Regulation 1924/2006 (a minimum decrease of 30% is needed). The decrease achieved was due to the significant changes made in the formulation of those ingredients that supplied lipids, proteins, and free sugars. The two animal origin ingredients, butter and milk, were totally substituted by an emulsion gel that vehiculized a vegetable oil rich in MUFAs (high oleic sunflower oil) and by a soy protein suspension, respectively.

Although differences between the control and the reformulated cakes were statically significant, the protein content was around 5 g/100 g of the product in both cases (5.1–5.3%). Both products were made with the same amount of wheat flour, and the added soy protein in the reformulated product was calculated to compensate for the protein supplied by milk in the control (around 1%). Proteins from wheat flour, especially gluten, are the main ones responsible for the network structure formed in the baking process. However, an excess of protein results in a harder structure as a consequence of the strong adherence between protein and starch [26,27].

Regarding the quantity of carbohydrates, it has to be pointed out that it was not possible to carry out this measurement in this work, so data shown in Table 2 were obtained by difference. Carbohydrates were supplied by wheat flour, sugar/polyols, and in the case of control products, the lactose found in the milk. Wheat flour basically contributes to the starch, which is involved in the development of the dough through its interaction with proteins. The same amount of starch and proteins in the reformulated products and the control ones is a key factor in the slight differences found in the textural properties between them. As refined flour was used, no significant amount of fiber was supposed to be found in the final product. In fact, in the nutritional information shown on the label, no data were found for fiber. Flour supplied 75% carbohydrates, including 4% sugars. The 8% of maltitol used in the reformulated products led to a lower energy supply in the reformulated product. The conversion factor, established by EU Regulation 1169/2011 for polyols, is 2.4 kcal/g, instead of 4 kcal/g for the rest of carbohydrates, so it means a reduction of 12.8 kcal/100 g as a consequence of the use of the sweetener [28]. Moreover, the lower amount of sugar added to the reformulated product made it possible to achieve a 36% reduction in this nutrient, which was enough to meet the standard for the claim of “reduced sugar”.

The main ingredient supplying fat in the reformulated products was the gelled emulsion, giving rise to 4.5% fat, which is more than a 67% decrease compared to the control. In this case, the 30% reduction needed for obtaining the fat reduction claim was widely achieved. It is true that the great debate over the actual implication of total fat intake in health still exists. The direct association between total fat intake and the risk of heart disease, diabetes, cancer, or adiposity has not been fully proven thus far [29]. However, it is a matter of fact that fat is the main energetic macronutrient and, when ingested in excess, it leads to an excess of energy intake, obesity, and related diseases. In any case, when elaborating bakery products, a certain amount of fat is necessary from technological and sensory points of view. During the mixing and beating of the ingredients, fat (due to its surface-active properties) contributes to incorporating and stabilizing gas bubbles in the dough and also prevents the excessive development of gluten proteins [14]. Fat also contributes to improving the final texture and increasing the volume [30].

Moreover, the changes in the nature of the ingredients caused interesting modifications in the lipid fractions of the reformulated cake. Table 3 reports the fatty acids profiles for both products. Trans fatty acids were very low in both products, being a bit higher in control products (0.3%), probably due to the presence of t11C18:1 among the C18:1 trans isomers, which is abundant in ruminant fats (butter) [31]. Low-medium chain and saturated fatty acids, typical from animal-origin fat as caproic, caprylic, capric, lauric, and myristic acids were not detected in the reformulated cake, reaching 3 g/100 g of the product in the control cake. This product also showed a high amount of palmitic acid (5.1 g/100 g cake), reaching a total SFA of 9.45%. On the contrary, the reformulated product had only 0.52% SFA, being susceptible to be claimed as “reduced in saturated fat”. The substantial health benefits that the replacement of saturated fats with unsaturated fats can confer have been extensively demonstrated with scientific evidence. The evaluation of the associations of specific dietary fats with total and cause-specific mortality in two large, ongoing cohort studies concluded that replacing 5% of energy from saturated fats with equivalent energy from PUFA (polyunsaturated fatty acids) and MUFA (monounsaturated fatty acids) was associated with important reductions in mortality [32]. No big differences were found in the amounts of MUFAs and PUFAs between reformulated and control cakes, so PUFAs/SFA and PUFAs + MUFAs/SFA ratios were much higher for the former ones. Although no measures of cholesterol were obtained experimentally in this work, we can hypothesize that this compound was not present in the reformulated products. Although the concern about the implications of cholesterol intake in cardiovascular health has decreased in the last years in favor of fatty acids nature, controlling its intake is still recommended [33].

### 3.2. Texture

From the consumer’s perspective, texture properties are of great importance in a cake. It has been reported that this feature is determined by the ratio of the combination of the main ingredients (sugar, butter, margarine, and eggs) [34]. In our work, hardness did not show statistical differences between control and reformulated products in spite of the higher moisture content of the reformulated ones. The evaluation of the influence of scarlet runner bean flour on the textural properties of vegan cakes containing 4.3–8.7% proteins showed hardness values between 71.63 and 124.33 N, which were much higher than those found in our products (3.087–3.035 N) [35]. However, the moisture of those vegan cakes was in the range of 6.6–7.1%, which was much lower than the moisture content of the reformulated product in this work (32%). It is worth noting that great variability can be found in the hardness values, even within the same type of products, depending on the ingredients used. Hardness in the range of 13–23.6 N for vegan muffins with 23–26% moisture and 4.3–4.5% proteins has been reported [36]. These authors found that hardness increased with fiber addition, which is in agreement with results obtained in enriched fiber muffins [35,37]. Pérez-Santana et al. [38], using high oleic palm oils in fluid shortenings containing 30% saturated fatty acids, found that the obtained shortenings had similar physical properties and did not negatively affect the texture of cookies.

### 3.3. Color

**Figure 1:**
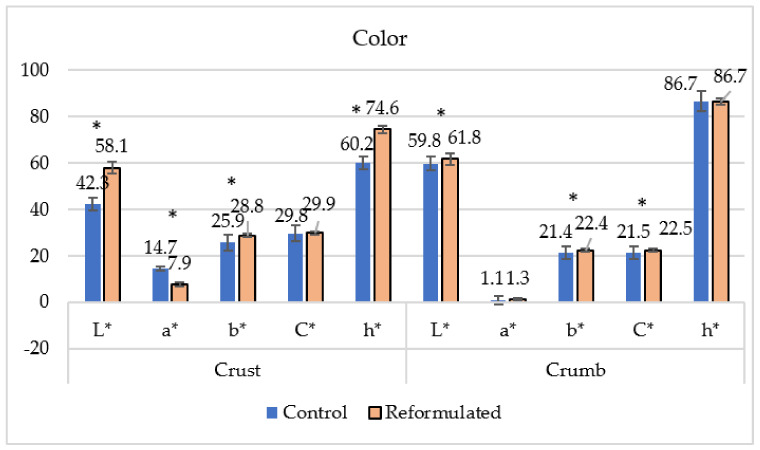
Color parameters of control and reformulated cakes. The results are expressed as mean values. The standard deviations are indicated using bars. Significant differences (*p* < 0.05) between control and reformulated cakes are indicated with an asterisk.

The color of the crust and the crumb was measured using the CieL*a*b* system (Figure 1). In general, reformulated bakery products elaborated with whole flours, intending to increase the fiber amount, showed great differences in color compared to control ones. Differences in CieL*a*b* measures for crust and crumb have also been reported in vegan muffins [35,36]. While crust color is affected by the Maillard reaction between reducing sugars and amino acids during baking, crumb color is influenced by cake ingredients [13]. In our work, the color of the crumb of both products was quite similar. The hue values did not show statistical differences for either of the a* (redness) values. It also has to be noted that the differences found for L* (brightness), b* (yellowness), and C*, although statistically significant, were really small. More relevant differences were found for crust color measures (See Figure S1, Supplementary materials). The L*, b*, and hue values were higher in reformulated products, whereas a* was higher in control products. The lighter color of the crust in the reformulated product could be due to the higher moisture content of this product. The similar baking conditions for both types of products should lead to a similar dehydration process [39], and thus, a higher amount of water was maintained in the reformulated product’s crust. All these results can be reflected in the Browning Index [24], which was calculated with the means of the CieL*a*b* measures. The results clearly showed that, although some differences could be observed in the crust (BI 110.46 control, 75.58 reformulated), the color of the crumb was very similar (BI 0.385 control, 0.387 reformulated).

### 3.4. Sensory Analysis

**Figure 2:**
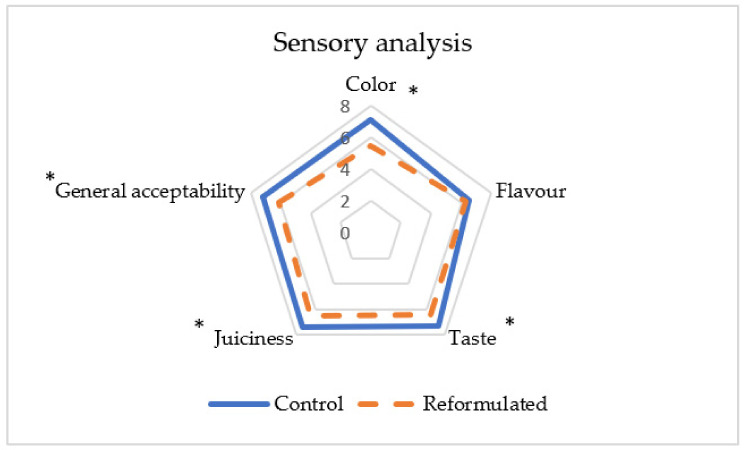
Sensory analysis of control and reformulated cakes. Significant differences (*p* < 0.05) between control and reformulated cakes are indicated with an asterisk.

Sensory analysis of the products showed that reformulated products did not obtain the same scores in color, taste, juiciness, and general acceptability as compared to the control formulation (Figure 2). However, all these properties were quite well evaluated, with scores over 6. Giuffrè et al. [40], reformulating Italian Cantuccini biscuits with extra virgin olive oil, also observed lower scores for every sensory parameter (color, taste, flavor, texture, and overall acceptability) as compared to the control biscuits. In our work, regarding taste, some of the panelists found control products sweeter than the reformulated ones; this is the cause for the lower score (6.4 for reformulated and 7.3 for control). As for juiciness, the scores were also slightly lower in the reformulated products (6.5 in reformulated and 7.4 in control), in spite of the fact that no hardness differences were found in the instrumental analysis. Results for color confirmed those obtained by CieL*a*b*, with slightly lower values for color (5.5 compared to 7.1 in the control), probably due to the lower color intensity of the reformulated cakes. For flavor, no significant differences were found between products. In general terms, obtaining reformulated products with a healthier nutritional profile while maintaining similar acceptability values as compared to regular formulations still remains a challenge [36,41,42]. In addition, when the replacement strategy tackles two nutrients at the same time (fat and sugar), more elaborated approaches are needed [43]. It is worth mentioning that, in previous works dealing with the sensory assessment of reformulated healthier foodstuffs, it has been stated that scores change (normally improving) when consumers are informed about the beneficial effects of the reformulated products prior to the sensory analysis [25,44,45].

## 4. Conclusions

Cakes elaborated without animal-origin ingredients could be a healthier and more sustainable option, showing acceptable sensory properties. The use of a gelled emulsion ingredient based on a high oleic sunflower oil at 20% and stabilized with alginate, as well as the incorporation of a soy protein suspension, seem to be useful and simple processes that could be implemented by the bakery industry to improve their products. In order to confirm these results, further studies at the industrial level should be carried out, including acceptability by potential consumers, especially among vegans.

## Figures and Tables

**Table 1:** Formulations of control and reformulated cakes.

Ingredients (g/100 g)	Control	Reformulated
Flour	32.3	32.3
Baking powder	1.4	1.4
Salt	0.3	0.3
Whole milk	27.6	0.0
Apple cider vinegar	1.5	1.5
Granulated sugar	23.2	15.0
Butter	13.2	0.0
Maltitol	0.0	8.0
Pure vanilla extract	0.5	0.5
Soy protein suspension (6%)	0.0	13.0
Gelled emulsion	0.0	28.0

**Table 2:** Nutritional composition of control and reformulated cakes.

Ingredients (g/100 g)	Control	Reformulated
Energy (kcal/100 g)	363	274
Moisture (g/100 g)	25.0 ± 0.5	32.0 ± 0.2 *
Lipids (g/100 g)	13.7 ± 0.0	4.5 ± 0.1 *
Carbohydrates ^1^ (g/100 g)	54.5	56.8
Sugars ^2^ (g/100 g)	27.2	17.5
Polyols ^2^ (g/100 g)	0.0	9.0
Protein (g/100 g)	5.3 ± 0.1	5.1 ± 0.1 *
Ash (g/100 g)	1.5 ± 0.1	1.6 ± 0.1

The results are expressed as mean values ± standard deviation. ^1^ Calculated by difference. ^2^ Calculated from the used ingredients. Significant differences (*p* < 0.05) between control and reformulated cakes are indicated with an asterisk.

**Table 3:** Fatty acids profile (g/100 g of product) of control and reformulated cakes.

Fatty Acid	Control	Reformulated
Caproic acid, C6:0	0.13 ± 0.01	ND
Caprylic acid, C8:0	0.13 ± 0.02	ND
Capric acid, C10:0	0.38 ± 0.07	ND
Lauric acid, C12:0	0.49 ± 0.06	ND
Myristic acid, C14:0	1.83 ± 0.10	ND
Palmitic acid, C16:0	5.10 ± 0.09	0.33 ± 0.003 *
t-Palmitoleic, C16:1	0.05 ± 0.004	ND
Palmitoleic acid, C16:1 n-7	0.27 ± 0.03	0.01 ± 0.001 *
Stearic acid, C18:0	1.42 ± 0.06	0.15 ± 0.003 *
∑Trans isomers, C18:1	0.27 ± 0.04	0.01 ± 0.002 *
Oleic acid, C18:1 n-9	2.92 ± 0.14	3.19 ± 0.02 *
Vaccenic acid, C18:1 n-7	0.10 ± 0.01	0.04 ± 0.001 *
t-Linoleic acid, C18:2	ND	ND
c-t Linoleic acid, C18:2	0.01 ± 0.001	ND
t-c Linoleic acid, C18:2	ND	ND
Linoleic acid, C18:2 n-6	0.49 ± 0.02	0.77 ± 0.005 *
Arachidic acid, C20:0	0.02 ± 0.002	0.01 ± 0.000
γ-linolenic acid, C18:3 n-6	ND	ND
Eicosenoic acid, C20:1 n-9	ND	ND
α-linolenic acid, C18:3 n-3	0.07 ± 0.01	0.02 ± 0.004 *
Eicosadienoic acid, C20:2 n-6	ND	0.01 ± 0.001
Behenic acid, C22:0	ND	0.04 ± 0.001 *
Brassidic acid, C22:1	0.01 ± 0.001	ND
Erucic acid, C22:1 n-9	ND	ND
Eicosatrienoic acid, C20:3 n-3	ND	ND
Arachidonic acid, C20:4 n-6	0.02 ± 0.002	0.01 ± 0.000 *
Eicosapentaenoic acid, C20:5 n-3	0.01 ± 0.001	0.01 ± 0.001 *
Nervonic acid, C24:1 n-9	ND	ND
Docosatrienoic acid, C22:3 n-9	ND	ND
Docosapentaenoic acid, C22:5 n-6	ND	ND
Lignoceric acid, C24:0	ND	ND
Docosapentaenoic acid, C22:5 n-3	ND	ND
Docosahexaenoic acid, C22:6 n-3	ND	ND
∑SFA	9.45 ± 0.35	0.52 ± 0.01 *
∑MUFA	3.17 ± 0.31	3.24 ± 0.02
∑PUFA	0.58 ± 0.05	0.81 ± 0.01 *
∑n3	0.08 ± 0.01	0.04 ± 0.001 *
∑n6	0.52 ± 0.02	0.77 ± 0.01 *
∑n6/n3	0.90 ± 0.06	0.90 ± 0.02
∑PUFA/SFA	0.01 ± 0.001	0.07 ± 0.002 *
∑PUFA + MUFA/SFA	0.05 ± 0.01	0.36 ± 0.01 *
∑Trans	0.31 ± 0.06	0.02 ± 0.002 *

The results are expressed as mean values ± standard deviation. ND, not detected. Significant differences (*p* < 0.05) between control and reformulated cakes are indicated with an asterisk.

## Data Availability

The date are available from the corresponding author.

## Supplementary Materials

The following supporting information can be downloaded at: https://www.mdpi.com/article/10.3390/foods12010054/s1, Figure S1: Aspect of Control and Reformulated cakes.

## References

[1] Raikos V., Ranawana V., Raikos V., Ranawana V. (2019). Reformulating Foods for Health-Concepts, Trends and Considerations. Reformulation as a Strategy for Developing Healthier Food Products.

[2] Garvey E.C., O’Sullivan M.G., Kerry J.P., Kilcawley K.N. (2020). Factors Influencing the Sensory Perception of Reformulated Baked Confectionary Products. Crit. Rev. Food Sci. Nutr..

[3] Kim J.N., Park S., Shin W.S. (2014). Textural and Sensory Characteristics of Rice Chiffon Cake Formulated with Sugar Alcohols Instead of Sucrose. J. Food Qual..

[4] Ronda F., Gómez M., Blanco C.A., Caballero P.A. (2005). Effects of Polyols and Nondigestible Oligosaccharides on the Quality of Sugar-Free Sponge Cakes. Food Chem..

[5] Estruch R., Vendrell E., Ruiz-León A.M., Casas R., Castro-Barquero S., Alvarez X. (2020). Reformulation of Pastry Products to Improve Effects on Health. Nutrients.

[6] Woodbury T.J., Grush E., Allan M.C., Mauer L.J. (2022). The Effects of Sugars and Sugar Alcohols on the Pasting and Granular Swelling of Wheat Starch. Food Hydrocoll..

[7] Hedayati S., Shahidi F., Koocheki A., Farahnaky A., Majzoobi M. (2016). Functional Properties of Granular Cold-Water Swelling Maize Starch: Effect of Sucrose and Glucose. Int. J. Food Sci. Technol..

[8] Struck S., Jaros D., Brennan C.S., Rohm H. (2014). Sugar Replacement in Sweetened Bakery Goods. Int. J. Food Sci. Technol..

[9] Lin S.D., Lee C.C., Mau J.L., Lin L.Y., Chiou S.Y. (2010). Effect of Erythritol on Quality Characteristics of Reduced-Calorie Danish Cookies. J. Food Qual..

[10] Gutiérrez-Luna K., Ansorena D., Astiasarán I. (2020). Flax and Hempseed Oil Functional Ingredient Stabilized by Inulin and Chia Mucilage as a Butter Replacer in Muffin Formulations. J. Food Sci..

[11] Koç M., Koç B., Susyal G., Yilmazer M.S., Ertekin F.K., Baǧdatlioǧlu N. (2011). Functional and Physicochemical Properties of Whole Egg Powder: Effect of Spray Drying Conditions. J. Food Sci. Technol..

[12] Yazici G.N., Ozer M.S. (2021). A Review of Egg Replacement in Cake Production: Effects on Batter and Cake Properties. Trends Food Sci. Technol..

[13] Hedayati S., Niakousari M., Seidi Damyeh M., Mazloomi S.M., Babajafari S., Ansarifar E. (2021). Selection of Appropriate Hydrocolloid for Eggless Cakes Containing Chubak Root Extract Using Multiple Criteria Decision-Making Approach. LWT.

[14] Peris M., Rubio-Arraez S., Castelló M.L., Ortolá M.D. (2019). From the Laboratory to the Kitchen: New Alternatives to Healthier Bakery Products. Foods.

[15] Willett W., Rockström J., Loken B., Springmann M., Lang T., Vermeulen S., Garnett T., Tilman D., DeClerck F., Wood A. (2019). Food in the Anthropocene: The EAT–Lancet Commission on Healthy Diets from Sustainable Food Systems. Lancet.

[16] Gutiérrez-luna K., Ansorena D., Astiasarán I. (2022). Use of Hydrocolloids and Vegetable Oils for the Formulation of a Butter Replacer: Optimization and Oxidative Stability. LWT.

[17] Horwitz W., AOAC (2002). Moisture in Meat. 950.46. Official Methods of Analysis.

[18] Horwitz W., AOAC (2002). Ash of Meat. 920.153. Official Methods of Analysis.

[19] Horwitz W., AOAC (2002). Nitrogen in Meat. Kjeldahl Method. 928.08. Official Methods of Analysis.

[20] (1973). Meat and Meat Products—Determination of Total Fat Content.

[21] Horwitz W., AOAC (2002). Fat in Flour. Acid Hydrolysis Method. 922.06. Official Methods of Analysis.

[22] Horwitz W., AOAC (2002). Methyl Esters of Fatty Acids in Oils and Fats. 969.33. Official Methods of Analysis.

[23] Ansorena D., Echarte A., Ollé R., Astiasarán I. (2013). 2012: No Trans Fatty Acids in Spanish Bakery Products. Food Chem..

[24] Ureta M.M., Olivera D.F., Salvadori V.O. (2014). Quality Attributes of Muffins: Effect of Baking Operative Conditions. Food Bioprocess Technol..

[25] Alejandre M., Astiasarán I., Ansorena D. (2019). Omega-3 Fatty Acids and Plant Sterols as Cardioprotective Ingredients in Beef Patties: Composition and Relevance of Nutritional Information on Sensory Characterization. Food Funct..

[26] Altindag G., Certel M., Erem F., Ilknur Konak Ü. (2015). Quality Characteristics of Gluten-Free Cookies Made of Buckwheat, Corn, and Rice Flour with/without Transglutaminase. Food Sci. Technol. Int..

[27] Moiraghi M., Vanzetti L., Bainotti C., Helguera M., León A., Pérez G. (2011). Relationship between Soft Wheat Flour Physicochemical Composition and Cookie-Making Performance. Cereal Chem..

[28] European Commission (2011). EC Regulation No 1169/2011, and Subsequent Modifications and Supplements. Off. J. Eur. Union.

[29] Willett W.C. (2011). The Great Fat Debate: Total Fat and Health. J. Am. Diet. Assoc..

[30] Rios R.V., Pessanha M.D.F., de Almeida P.F., Viana C.L., da Silva Lannes S.C. (2014). Application of Fats in Some Food Products. Food Sci. Technol..

[31] Raes K., Fievez V., Chow T.T., Ansorena D., Demeyer D., De Smet S. (2004). Effect of Diet and Dietary Fatty Acids on the Transformation and Incorporation of C18 Fatty Acids in Double-Muscled Belgian Blue Young Bulls. J. Agric. Food Chem..

[32] Wang D.D., Li Y., Chiuve S.E., Stampfer M.J., Manson J.A.E., Rimm E.B., Willett W.C., Hu F.B. (2016). Association of Specific Dietary Fats with Total and Cause-Specific Mortality. JAMA Intern. Med..

[33] Du X., Xin H. (2021). Association between Cholesterol Intake and All-Cause Mortality: NHANES-Linked Mortality Study. Cent. Eur. J. Public Health.

[34] Pancharoen S., Leelawat B., Vattanakul S. (2019). Using Texture Properties for Clustering Butter Cake from Various Ratios of Ingredient Combination. J. Food Meas. Charact..

[35] Göksel Saraç M., Dedebaş T., Hastaoğlu E., Arslan E. (2022). Influence of Using Scarlet Runner Bean Flour on the Production and Physicochemical, Textural, and Sensorial Properties of Vegan Cakes: WASPAS-SWARA Techniques. Int. J. Gastron. Food Sci..

[36] Bianchi F., Cervini M., Giuberti G., Rocchetti G., Lucini L., Simonato B. (2022). Distilled Grape Pomace as a Functional Ingredient in Vegan Muffins: Effect on Physicochemical, Nutritional, Rheological and Sensory Aspects. Int. J. Food Sci. Technol..

[37] Heo Y., Kim M.J., Lee J.W., Moon B.K. (2019). Muffins Enriched with Dietary Fiber from Kimchi By-Product: Baking Properties, Physical–Chemical Properties, and Consumer Acceptance. Food Sci. Nutr..

[38] Perez-santana M., Cagampang G.B., Nieves C., Cedeño V., Macintosh A.J. (2022). Use of High Oleic Palm Oils in Fluid Shortenings and Effect on Physical Properties of Cookies. Foods.

[39] Ureta M.M., Olivera D.F., Salvadori V.O. (2017). Influence of Baking Conditions on the Quality Attributes of Sponge Cake. Food Sci. Technol. Int..

[40] Giuffrè A.M., Caracciolo M., Capocasale M., Zappia C., Poiana M. (2022). Effects of Shortening Replacement with Extra Virgin Olive Oil on the Physical—Chemical—Sensory Properties of Italian Cantuccini Biscuits. Foods.

[41] Rahmati N.F., Mazaheri Tehrani M. (2015). Replacement of Egg in Cake: Effect of Soy Milk on Quality and Sensory Characteristics. J. Food Process. Preserv..

[42] Jarpa-Parra M., Wong L., Wismer W., Temelli F., Han J., Huang W., Eckhart E., Tian Z., Shi K., Sun T. (2017). Quality Characteristics of Angel Food Cake and Muffin Using Lentil Protein as Egg/Milk Replacer. Int. J. Food Sci. Technol..

[43] Richardson A.M., Tyuftin A.A., Kilcawley K.N., Gallagher E., O’sullivan M.G., Kerry J.P. (2021). The Application of Pureed Butter Beans and a Combination of Inulin and Rebaudioside a for the Replacement of Fat and Sucrose in Sponge Cake: Sensory and Physicochemical Analysis. Foods.

[44] Henrique N.A., Deliza R., Rosenthal A. (2015). Consumer Sensory Characterization of Cooked Ham Using the Check-All-That-Apply (CATA) Methodology. Food Eng. Rev..

[45] Grasso S., Harrison S.M., Monahan F.J., Brayden D., Brunton N.P. (2017). The Effect of Plant Sterol-Enriched Turkey Meat on Cholesterol Bio-Accessibility during in Vitro Digestion and Caco-2 Cell Uptake. Int. J. Food Sci. Nutr..

