# Supplementary figures and images for: A Cake Made with No Animal Origin Ingredients: Physical Properties and Nutritional and Sensory Quality
Source: Foods. 2022 Dec 22;12(1):54. doi: 10.3390/foods12010054 (PMC9818566; doi:10.3390/foods12010054)

CONTROL CAKE

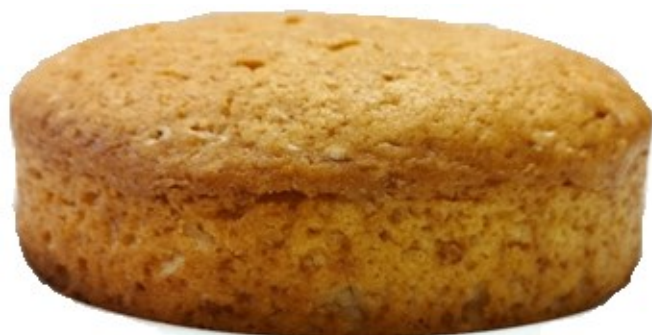

REFORMULATED CAKE

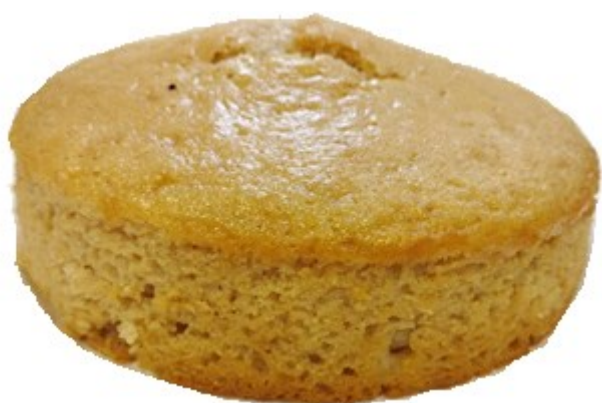

Figure S1. Aspect of Control and Reformulated cakes.

Supplement: Supplementary file 1 [file foods-12-00054-s001.zip › foods-2054904-supplementary.pdf]
